# Circadian Rhythm Disruption Influenced Hepatic Lipid Metabolism, Gut Microbiota and Promoted Cholesterol Gallstone Formation in Mice

**DOI:** 10.3389/fendo.2021.723918

**Published:** 2021-10-21

**Authors:** Chuanqi He, Weiyi Shen, Chaobo Chen, Qihan Wang, Qifan Lu, Wentao Shao, Zhaoyan Jiang, Hai Hu

**Affiliations:** Center of Gallstone Disease, Shanghai East Hospital, Institution of Gallstone Disease, School of Medicine, Tongji University, Shanghai, China

**Keywords:** circadian rhythm, gallstone, cholesterol, bile acid, gut microbiota

## Abstract

**Background:**

Hepatic lipid metabolism regulates biliary composition and influences the formation of cholesterol gallstones. The genes *Hmgcr* and *Cyp7a1*, which encode key liver enzymes, are regulated by circadian rhythm-related transcription factors. We aimed to investigate the effect of circadian rhythm disruption on hepatic cholesterol and bile acid metabolism and the incidence of cholesterol stone formation.

**Methods:**

Adult male C57BL/6J mice were fed either a lithogenic diet (LD) only during the sleep phase (time-restricted lithogenic diet feeding, TRF) or an LD *ad libitum* (non-time-restricted lithogenic diet feeding, nTRF) for 4 weeks. Food consumption, body mass gain, and the incidence of gallstones were assessed. Circulating metabolic parameters, lipid accumulation in the liver, the circadian expression of hepatic clock and metabolic genes, and the gut microbiota were analyzed.

**Results:**

TRF caused a dysregulation of the circadian rhythm in the mice, characterized by significant differences in the circadian expression patterns of clock-related genes. In TRF mice, the circadian rhythms in the expression of genes involved in bile acid and cholesterol metabolism were disrupted, as was the circadian rhythm of the gut microbiota. These changes were associated with high biliary cholesterol content, which promoted gallstone formation in the TRF mice.

**Conclusion:**

Disordered circadian rhythm is associated with abnormal hepatic bile acid and cholesterol metabolism in mice, which promotes gallstone formation.

## Introduction

Gallstone disease is one of the most common gastrointestinal diseases in Western countries (1), with an incidence of ~20% in adults (2). Almost 90% of gallstones are of the cholesterol type (2). The formation of cholesterol gallstones is a complex process that involves interactions between genetic and environmental factors (3). The liver is principally responsible for the regulation of biliary lipid and cholesterol concentrations, which is a key determinant of the formation of cholesterol gallstones (4–6).

Accumulating epidemiological evidence suggests that circadian disruption caused by shift work, jet lag, or sleep disorders is associated with metabolic diseases, such as obesity and metabolic syndrome (7, 8). Irregular eating or eating at the wrong time of day, including skipping breakfast and eating late at night, may contribute to metabolic disorders by disrupting the normal circadian rhythm (9). Animal studies have shown that time-restricted feeding affects body mass, adiposity, and other metabolic parameters (10–17). Mice fed a high-fat diet only during the sleep (light) phase over a period of 6 weeks gained 2.5-fold more weight than mice fed the same diet during the active (dark) phase, despite the energy intake and physical activity of the groups being identical (18).

The expression of genes that encode key enzymes in hepatic bile acid and cholesterol metabolism, such as *Hmgcr* and *Cyp7a1*, is regulated by circadian rhythm-related transcription factors (19). An internal circadian timing system entrains various physiological and behavioral rhythms, such as sleep-wake cycles, metabolism, and hormone secretion (20). The mammalian master clock is located in the suprachiasmatic nucleus (SCN) of the anterior hypothalamus (21) and is driven by core clock genes, such as *Clock*, *Bmal1*, *Per1*/*2*, and *Cry1/2*, through transcriptional feedback loops (21). *Clock* and *Bmal1* form heterodimers to activate target genes, including *Per* and *Cry*, by binding to E-box elements in their promoters (21). The heterodimerized PER and CRY proteins translocate to the nucleus, where they inhibit CLOCK : BMAL1 transcriptional activity (21). Circadian oscillators that are referred to as peripheral clocks are located in most peripheral organs, including the liver, heart, lungs, skeletal muscle, and adipose tissue (21). These peripheral clocks are synchronized to the SCN by systemic time cues, including neuronal signals and circulating humoral factors such as glucocorticoids and insulin (20). Components of the molecular clock regulate the expression of hundreds of metabolic output genes in peripheral tissues (20). Consequently, cholesterol and bile acids are metabolized rhythmically. Bile acid synthesis peaks at night in mice, during their activity period, and is low during the day, during their rest period. In humans, who are diurnal animals, the circadian rhythm of bile acid metabolism differs from that of mice, with two peaks at 13:00 [Zeigeiber time (ZT)7] and 21:00 (ZT15) (22). Cholesterol absorption and synthesis also display circadian rhythms, and the circadian variation in both cholesterol synthesis and absorption may be, at least in part, determined by the timing of food ingestion.

In recent years, the gut microbiota has been shown to play a key role in metabolic disease, and interestingly, the composition of the gut microbiota also shows circadian fluctuations. Effects of eating patterns on *Bacteroidetes* and *Firmicutes* may have a substantial influence on circadian rhythms in the gut, which would in turn regulate systemic metabolism (23). However, the specific mechanisms of such regulation remain to be determined. Periodic changes in bacterial metabolites may affect the host. For example, choline in food can be converted to trimethylamine and eventually trimethylamine N-oxide, which affects the expression of *Clock* and *Bmal1* in endothelial cells (24). Rhythm-induced changes in the microbiota also play a role in the development of tumors. Disruption in the food-intake phase reduces the abundance of short-chain fatty acid-producing bacteria, which affects the butyric acid metabolism pathway, and ultimately contributes to the development of colon cancer (25). Moreover, bacteria activate pattern recognition receptors, including NOD-like receptors and toll-like receptors. Activation of these receptors on intestinal epithelial cells and immune cells alter the metabolic status of the host, which may predispose toward metabolic disease (26).

In a preliminary study, we identified enrichment of genes involved in circadian rhythms in the livers of mice fed an LD by RNA-seq analysis. This suggested that disturbance of hepatic circadian rhythms may play an important role in the promotion of gallstone formation. In the present study, we altered the circadian rhythm of mice through restricted feeding, and identified effects on hepatic cholesterol and bile acid metabolism, and the gut microbiota, which were associated with a higher risk of gallstone formation.

## Materials and Methods

### Study Design and Animal Experiment

Six-week-old, male C57BL/6J mice were purchased from Shanghai Model Organisms (Shanghai, China). After acclimatization to their environment, the mice were randomly allocated to groups that were fed an LD containing 1.25% cholesterol and 0.5% cholic acid only during the sleep phase (ZT0–12) (TRF) or ad libitum (nTRF) for 4 weeks. Water was available ad libitum. The energy intake of the mice was determined by providing pre-weighed food and weighing the leftover food each week. The body mass of the mice was measured twice a week. The mice were anesthetized with Avertin and sacrificed at 4-hour intervals, then their tissues were collected, weighed, rapidly frozen in liquid nitrogen, and then stored at −80°C until analysis. The Institutional Animal Care and Use Committee approved all the experimental protocols.

### Lipid Analysis

The serum cholesterol and triglyceride concentrations were determined using enzymatic methods (Applygen E1015, KHB Triglyceride kit). Biliary lipids were extracted using the Folch method and analyzed using an enzymatic methods, as previously described (27). The presence of crystals or gallstones was scored as previously described (28). Briefly, the presence of gallstones was identified visually by holding the gallbladder up against a light. A score of “++” referred to “around ten fine crystals were found”.

### Determination of Gene Expression by Quantitative Real-Time PCR

RNA was extracted from liver samples using TRIzol reagent (Invitrogen, Carlsbad, CA, USA), then single-stranded cDNA was synthesized using PrimeScript™ RT reagent kits and gDNA Eraser (Takara Bio). Real-time PCR was then performed using SYBR^®^ Premix Ex Taq™ II (Takara Bio) and an ABI QuantStudio6 Q6 (Thermo Fisher Scientific Inc., USA), with the sequences shown in **Supplementary Table 1**. The expression of each target gene was normalized to that of GAPDH and calculated using the delta Ct method.

### Analysis of the Gut Microbiota

The cecal contents of each mouse were collected at sacrifice and the microbial DNA was extracted, and then appropriate primers were used to amplify unique regions of the 16S rRNA. Next generation sequencing was performed, and the sequences obtained were classified into operational taxonomic units when they showed 97% similarity. The alpha- and beta-diversity of the microbiota were calculated, and the filtered data were compared to an existing database. The details of the sequencing have been described previously (29).

### Statistics

All data are expressed as mean ± standard error of the mean (SEM). Body mass, tissue mass, cumulative food consumption, and food efficiency data were compared between groups using Student’s t-test. Analyses were performed using SPSS 20.0 and *P* < 0.05 was taken to indicate a statistically significant difference.

## Results

### Time-Restricted Lithogenic Diet-Feeding (TRF) Promotes Gallstone Formation

**Figure 1:**
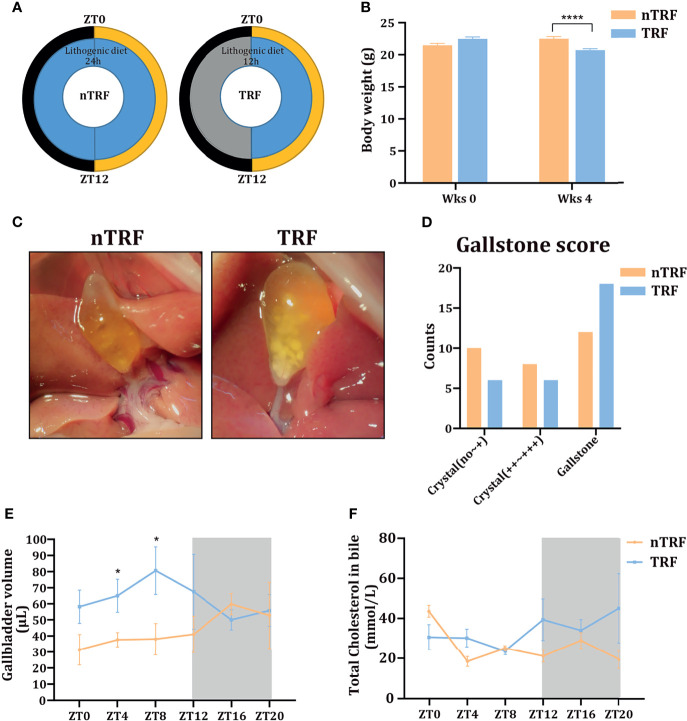
Time-restricted lithogenic diet-feeding (TRF) promotes gallstone formation. **(A)** Schematic diagram of the animal experiment. **(B)** Body masses of the mice at the beginning and the end of 4 weeks of feeding. **(C)** Appearance of the gallbladder in non-TRF (left) and TRF (right) mice. **(D)** The overall evaluation of gallstone. **(E)** Gallbladder volume of mice in the TRF and non-TRF group at various Zeitgeber times (ZT). **(F)** Biliary cholesterol concentrations at various ZT. Data are expressed as mean ± SEM (n = 5 mice/group at each time point). “*”means p < 0.05, “****” means p < 0.0001.

A schematic diagram of animal experiment is shown in **Figure 1A**. The groups of mice consumed equivalent amounts of energy. Mice with TRF gained slightly more weight than mice that underwent non-time-restricted lithogenic diet feeding (nTRF) over a 4-week period (**Figure 1B**).

The overall incidence of gallstones was more in TRF group compared with nTRF group (**Figures 1C, D**). The overall volume of the gallbladder was higher in the TRF group than in the nTRF group, especially at ZT0, 4, 8, and 12 (**Figure 1E**), as was the biliary cholesterol content at ZT12, 16, and 20 (**Figure 1F**).

### Influences of Circadian Rhythm on Serum Lipid Concentrations

**Figure 2:**
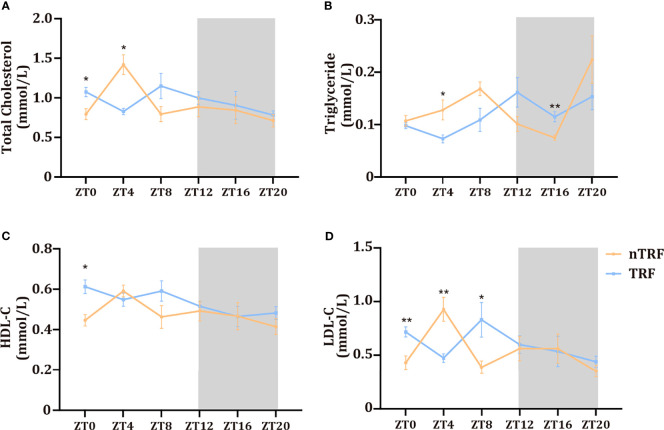
Time-restricted lithogenic diet-feeding (TRF) influences serum lipid concentrations. Serum total cholesterol **(A)**, triglyceride **(B)**, HDL **(C)**, and LDL **(D)** concentrations at various ZT. Data are expressed as mean ± SEM (n = 5 mice/group at each time point). “*” means p < 0.05, “**” means p < 0.01.

Changes in the circadian rhythm affected serum lipid concentrations. The peaks and troughs of total cholesterol and low-density lipoprotein (LDL)-cholesterol were almost opposite in their timing in the two groups. However, the high-density-lipoprotein (HDL)-cholesterol rhythm did not differ. The triglyceride (TG) peak in the TRF group was delayed by ~4 hours (**Figure 2**).

### TRF Disturbs the Hepatic Circadian Rhythm

**Figure 3:**
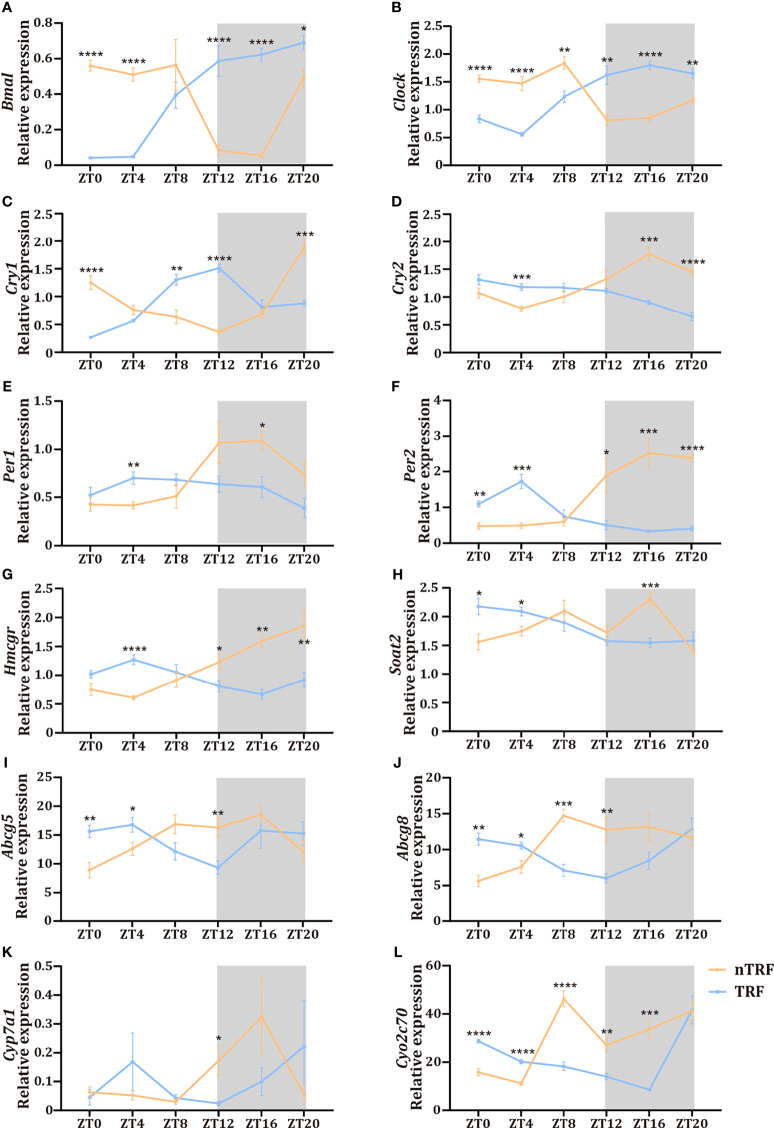
Time-restricted lithogenic diet-feeding (TRF) disrupts the expression patterns of hepatic genes. **(A–F)** Expression of hepatic central clock genes at various ZT. **(G–L)** Expression of genes involved in cholesterol and bile acid metabolism at various ZT. Data are expressed as mean ± SEM (n = 5 mice/group at each time point). “*” means p < 0.05, “**” means p < 0.01, “***” means p < 0.001, “****” means p < 0.0001.

The dietary pattern affected the dynamics of the RNA expression of circadian-associated genes in the liver. The patterns of expression of the key circadian genes *Clock* and *Bmal* were reversed by TRF (**Figures 3A, B**), and the normal circadian rhythms of *Per1/2/3* and *Cry1/2* expression were lost in the TRF group (**Figures 3C–F**).

### TRF Induces Disturbances in the Circadian Rhythms of Hepatic Lipid Metabolism

Hepatic cholesterol and bile acid metabolism are key determinants of biliary cholesterol content, and therefore gallstone formation. We found significant circadian fluctuations in genes involved in cholesterol synthesis (*Hmgcr*), cholesterol esterification (*Soat2*), canalicular cholesterol transport (*Abcg5* and *Abcg8*), and bile acid synthesis (*Cyp7a1*, *Cyp2c70*) (**Figures 3G–L**) in the nTRF group. These gene expression patterns were significantly altered by TRF, and manifested as reversals of the rhythms, decrease the expression peak and the narrowing of the amplitudes. Notably, the peak level of expression of *Cyp7a1* was significantly lower and the expression of *Soat2* decreased progressively after ZT8 in the TRF group.

### Effects of TRF on the Circadian Rhythmicity of the Gut Microbiota

**Figure 4:**
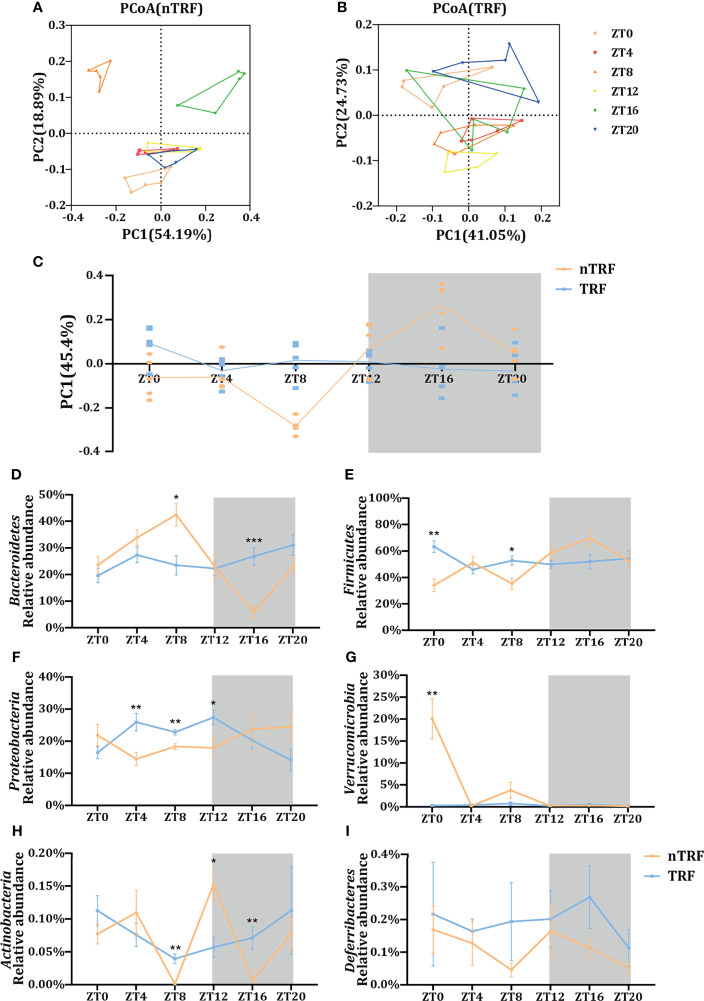
Effects of time-restricted lithogenic diet-feeding (TRF) on the gut microbiota. PCoA (weighted unifrac) analysis of the gut microbiota of mice in the non-TRF **(A)** and TRF **(B)** groups. **(C)** Differences in PC1 (45.4%) of the PCoA in mice from non-TRF and TRF groups. The line represents the mean value for each group. **(D–I)** Relative abundances of the six most common bacterial taxa in the non-TRF and TRF groups at various ZT. Data are expressed as mean ± SEM (n = 5 mice/group at each time point). “*” means p < 0.05, “**” means p < 0.01, “***” means p < 0.001.

The cecal contents of the mice were collected for the analysis of the gut microbiota by 16S rRNA gene sequencing. The microbial composition fluctuated over time in the nTRF group. In contrast, this fluctuation was almost lost in the TRF group (**Figure 4C**). Principal co-ordinates analysis (PCoA) showed a separation of the gut microbial distribution according to diet and time (**Figures 4A, B**). Differences at the phylum level were identified between the TRF and nTRF groups (**Figures 4D–I**). Compared with the nTRF group, the gut microbiota in the TRF group showed less fluctuation and no clear rhythm, especially with regard to *Bacteroidetes* and *Firmicutes*, which were the two most abundant phyla (**Figures 4D, E**).

## Discussion

In the present study, we found that TRF altered the circadian rhythms of and promoted gallstone formation in mice. This was associated with disruptions in hepatic cholesterol and bile acid metabolism and the gut microbiota, which exacerbated abnormalities in serum and biliary cholesterol concentrations.

Accumulating evidence suggests that chronic jet lag, irregular meal times, and the consumption of high-energy food (e.g., sugars) cause circadian dysfunction and cause the progression of metabolic diseases, such as obesity (30), diabetes (31), atherosclerosis (32), and hyperlipidemia (33). Links between disorders in circadian rhythms and gallstone disease have also been suggested: people who have lifestyles characterized by rhythm disorders, such as those who undertake frequent night work, participate in night-time entertainment and food consumption, or undertake prolonged diurnal shifts, are at higher risk of developing gallstone disease (34).

Mice are nocturnal animals that usually eat more during the night, when they are active. Both the central and peripheral clocks can be reset by environmental timing factors, also known as “zeitgebers”. The central clock is primarily set by light, while peripheral metabolic organs, such as the liver, are more sensitive to factors such as meal timing and the energy obtained from food. One study has shown that feeding a high-fat diet during the light period exacerbates the effects of this diet on mice, compared with those fed the same amount of food only during the dark period (18). In the present study, we changed the eating pattern of mice by restricting the availability of food to between ZT0 and 12 alone, during the day (the rest period). This TRF appeared to alter the circadian rhythms of the mice. The patterns of expression of *Clock* and *Bmal* were temporally reversed, and the expression of the *Per1* and *Cry2* genes lost its rhythmicity. In the TRF group, the pattern of expression of *BMAL1*, which is an important part of peripheral circadian rhythms (35), was reversed.

The circadian rhythm is regulated by clock genes, the dysregulation of which affects genes that mediate hepatic cholesterol and bile acid metabolism (36). We have shown that such dysregulation predisposes toward cholesterol gallstone formation in mice. To our knowledge, this was the first study to investigate how circadian rhythm disturbances lead to abnormal cholesterol and bile acid metabolism, and thereby gallstone formation.

Cholesterol synthesis peaks during the night in normal rodents, largely because they are nocturnal, and the peak coincides with feeding activity. Edwards et al. (37) showed an abnormal rhythm in the expression of *Hmgcr*, which encodes the key enzyme in cholesterol synthesis, after mice were administered acetate away from their normal feeding time. This is consistent with the present finding that the peak *Hmgcr* expression in the TRF group was during the light phase and that in the nTRF group was during the dark phase. The metabolism of bile acids is also rhythmic, with the peak occurring at the same time as cholesterol metabolism. The liver is the main site for the conversion of cholesterol to bile acids, which is regulated by nuclear receptors such as FXR, FGF15, and SHP, the expression of which fluctuates in a circadian rhythm (38). *Cyp7a1*, which encodes a key enzyme in bile acid synthesis, is regulated by a variety of circadian-related pathways. Furthermore, *Cyp2c70*, which generates β-muricholic acid, a hydrophilic bile acid that increases the solubility of cholesterol in bile, is regulated in a circadian fashion (39).

In the present study, the expression of genes involved in hepatic cholesterol and bile acid metabolism showed clear rhythmicity in the nTRF group, but this was significantly different in the TRF group. Specifically, the rhythms were reversed and the expression peaks were lower and narrower. The expression of *Soat2* in the TRF group continued to decrease after ZT8 and the peak expression of *Cyp7a1* was significantly lower than that of the nTRF group. Furthermore, the overall expression of *Cyp2c70* decreased in the TRF group within 24 h. These differences suggest that TRF causes the livers of mice to convert less free cholesterol to cholesteryl esters and cholesterol to bile acids than in nTRF mice. The resulting excess cholesterol would be secreted into bile in larger quantities, promoting gallstone formation.

We also found that the dietary pattern affected the gut microbiota. For example, the fluctuation in the abundance of *Bacteroidetes* was abolished by TRF. A lack of *Bacteroidetes* and *Firmicutes* is thought to contribute to the metabolic syndrome (40), but it is unclear whether the loss of the fluctuation in the abundance of this phylum may have been involved in the metabolic disorder identified. Recently, bile salt hydrolase (BSH) activity was shown to be lower in patients who had a low *Bacteroidetes*/*Firmicutes* ratio (41). The gut microbiota of the TRF mice had lower BSH activity, which would have reduced the conversion of conjugated bile acids to free bile acids, which have lower water solubility, resulting in an increase in the size of the bile acid pool and cholesterol deposition.

Recent studies have shown that rectifying the circadian rhythm by restricting the duration of the feeding period, without affecting the amount of food intake, ameliorates the metabolic syndrome in mice (42). Furthermore, time-restricted feeding improves the metabolic status of patients with obesity and prediabetes (43). A study of mice with a genetic mutation in their biological clock showed that the restriction of high-fat diet consumption to the activity period restores metabolic rhythms (42) and prevents metabolic diseases, such as fatty liver, hyperlipidemia, and diabetes (44). This suggests that the promotion of time-restricted eating in individuals with impaired circadian rhythms, such as shift workers, may improve their metabolism and prevent metabolic diseases. However, further research is needed to determine whether time-restricted eating can prevent gallbladder stones.

The present study had some limitations. First, we did not assess effect on SCN under our experimental condition. In our study, apparent alteration of circadian rhythm in liver was found and it suggested that such alteration was enough to change the hepatic metabolism related with cholesterol gallstone formation. Second, the circadian rhythm of mice is opposite to that of humans, therefore, the applicability of the present findings needs further confirmation on humans.

## Conclusion

We have provided evidence that TRF causes alterations in the circadian rhythms of mice, which lead to disorders in hepatic cholesterol and bile acid metabolism and the gut microbiota, which would promote the formation of cholesterol gallstones. These data suggest the importance of regular behavioral and eating habits in the prevention of cholelithiasis.

## Data Availability Statement

The datasets presented in this study can be found in online repositories. The names of the repository/repositories and accession number(s) can be found below: NCBI SRA BioProject, accession no: PRJNA765885.

## Ethics Statement

The animal study was reviewed and approved by Tongji University Institutional Animal Care and Use Committee.

## Author Contributions

CH and WYS are responsible for manuscript writing. CC, QW, QL, and WTS are responsible for experiments. ZJ and HH are responsible for the experiment design. All authors contributed to the article and approved the submitted version.

## Funding

This study was supported by grants from the National Natural Science Foundation of China (nos. 81770625 and 81770626) and the Key Specialty Construction Project of the Pudong Health and Family Planning Commission of Shanghai (no. PWZzk2017-10).

## Conflict of Interest

The authors declare that the research was conducted in the absence of any commercial or financial relationships that could be construed as a potential conflict of interest.

## Publisher’s Note

All claims expressed in this article are solely those of the authors and do not necessarily represent those of their affiliated organizations, or those of the publisher, the editors and the reviewers. Any product that may be evaluated in this article, or claim that may be made by its manufacturer, is not guaranteed or endorsed by the publisher.
