# Supplementary figures and images for: Circadian Rhythm Disruption Influenced Hepatic Lipid Metabolism, Gut Microbiota and Promoted Cholesterol Gallstone Formation in Mice
Source: Front Endocrinol (Lausanne). 2021 Oct 21;12:723918. doi: 10.3389/fendo.2021.723918 (PMC8567099; doi:10.3389/fendo.2021.723918)

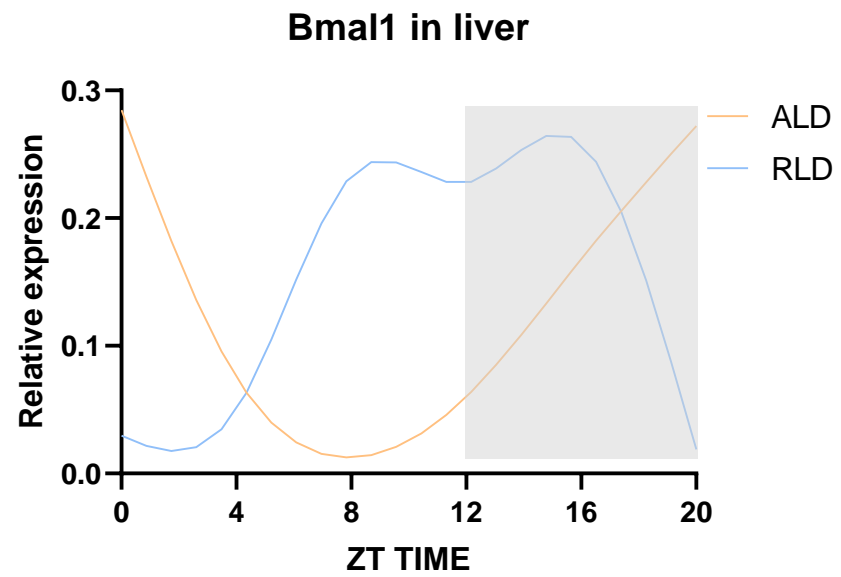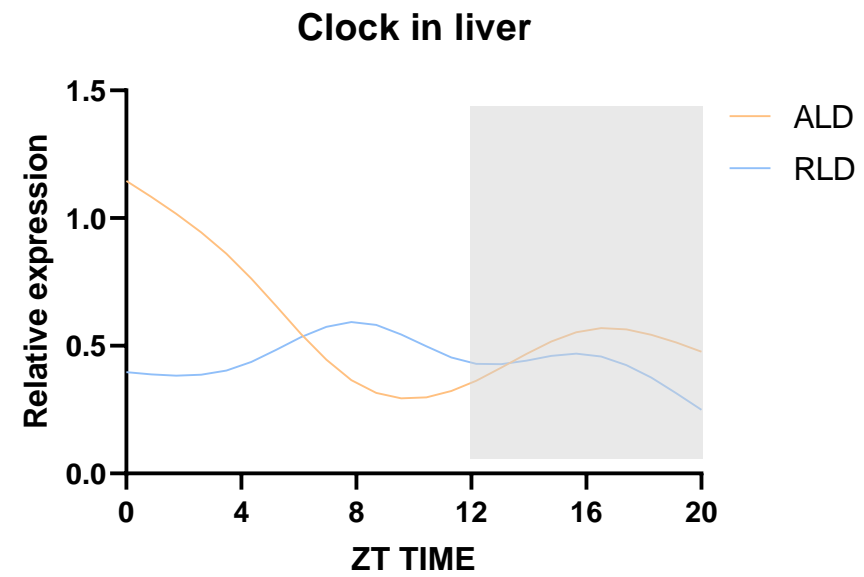

Supplement: Supplementary file 1 [file DataSheet_1.pdf]

**Bmal1 in hypothalamus**

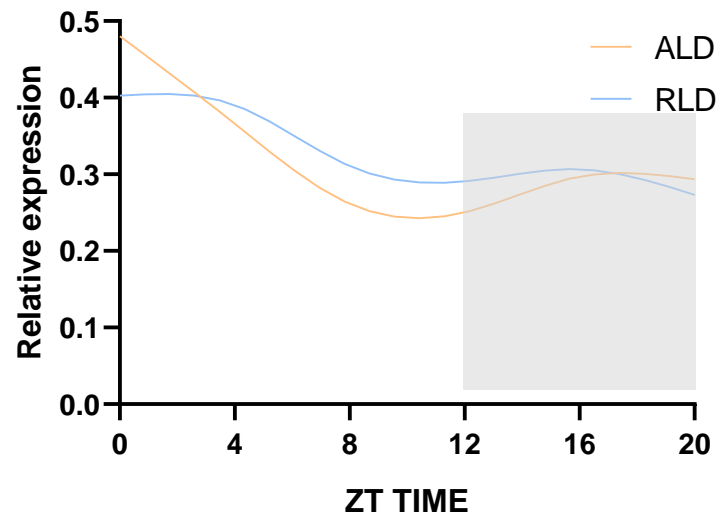

**Clock in hypothalamus**

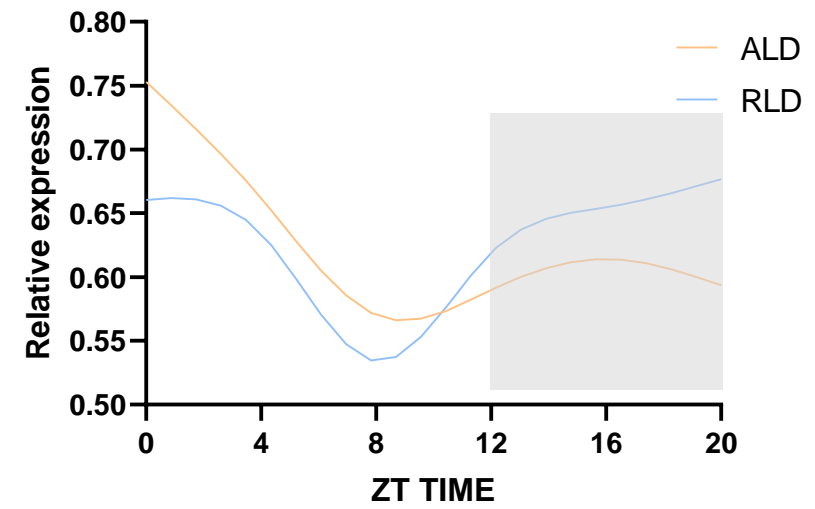

Supplement: Supplementary file 2 [file DataSheet_2.pdf]
